# Patient safety during final-year clerkships: A qualitative study of possible error sources and of the potential of Entrustable Professional Activities

**DOI:** 10.3205/zma001226

**Published:** 2019-03-15

**Authors:** Anja Czeskleba, Ylva Holzhausen, Harm Peters

**Affiliations:** 1 Charité – Universitätsmedizin Berlin, Dieter Scheffner Fachzentrum für medizinische Hochschullehre und Ausbildungsforschung, Prodekanat für Studium und Lehre, Berlin, Germany

**Keywords:** Final-year clerkships, patient safety, Entrustable Professional Activities

## Abstract

**Aim: **In final-year clerkships, such as the Practical Year in Germany, students’ workplace learning has to be balanced with the ensuring of patient safety. In this qualitative study, we investigated problems concerning patient safety as perceived from the perspective of supervising physicians, and whether and to what extent Entrustable Professional Activities (EPAs) can lead to an improvement in patient safety.

**Method: **Data was collected through focus groups. Participants were specialist physicians with experience of final-year clerkship training (n=11). The analysis of problems influencing patient safety was carried out deductively with an existing system of categories (error factors in the clinic). To identify potential improvements through EPAs, an inductively developed category system on the influence of EPAs in final-year clerkships was used.

**Results: **Supervising physicians perceive a variety of problems which affect patient safety. These can be found in the categories organization and management, individual factors, task factors and work environment. The physicians feel that EPAs may lead to an improvement in training and subsequently in patient safety. Their comments can be collated to the categories improvement in training, performance levels and supporting learning processes, transparency and minimizing uncertainty.

**Conclusions: **Statements by supervising physicians indicate a variety of problems in patient safety during the training of final-year clerkship students, for instance the lack of structure to the training. In their view, the implementation of EPAs can substantially reduce such risks, as they provide better content and organizational structure to the final-year clerkship.

## Zusammenfassung

**Zielsetzung: **In Final Year Clerkships, wie dem Praktischen Jahr (PJ), gilt es, eine Balance zwischen dem praxisbezogenen Lernen der PJ-Studierenden und der Gewährleistung der Patientensicherheit zu finden. In dieser qualitativen Studie wird aus Sicht von supervidierenden Ärzten exploriert, welche Probleme im PJ hinsichtlich der Patientensicherheit wahrgenommen werden und ob und inwiefern Entrustable Professional Activities (EPAs) zu einer Verbesserung der Patientensicherheit führen können.

**Methodik: **Die Datenerhebung erfolgte mittels Fokusgruppen. Teilnehmende waren in der PJ-Ausbildung erfahrene Fachärzte (n=11). Die Auswertung bzgl. vorhandener Probleme im PJ mit Einfluss auf die Patientensicherheit erfolgte deduktiv mit einem bereits vorliegenden Kategoriensystem (Fehlerfaktoren in der Klinik). Zur Analyse potentieller Verbesserungen durch EPAs wurde ein induktiv am Material entwickeltes Kategoriensystem zum Einfluss von EPAs auf das PJ genutzt.

**Ergebnisse: **Die supervidierenden Ärzte nehmen im PJ eine Reihe von Problemen mit Einfluss auf die Patientensicherheit wahr. Diese lassen sich den Kategorien Organisation & Management, individuelle Faktoren, Aufgabenfaktoren und Arbeitsumfeld zuordnen. Nach ihrer Einschätzung könnten EPAs zu einer Verbesserung der Ausbildung und damit verbunden der Patientensicherheit beitragen. Ihre Kommentare lassen sich den Kategorien Verbesserung der Ausbildung, Leistungsstand & Unterstützung von Lernprozessen, Transparenz und Minimierung von Unsicherheit zuordnen.

**Schlussfolgerungen: **Die Aussagen der Ärzte zeigen verschiedene Probleme in der Patientensicherheit bei der Ausbildung von PJ-Studierenden auf, bspw. durch eine mangelnde Strukturierung des PJ. Ihrer Einschätzung nach kann das Risiko durch den Einsatz von EPAs maßgeblich reduziert werden, da sie das PJ inhaltlich und organisatorisch besser strukturieren.

## 1. Introduction

In many countries, undergraduate medical education ends with a practical section, also known as final-year clerkships. During these clerkships, students are actively involved in patient care. They apply their knowledge, mainly acquired theoretically during their studies, in a practical setting, and are prepared for their future work being physicians. As students train, it is important to find a balance between their learning and the safety of patients [1]. One way this is ensured is that students work under the supervision of physicians, and moreover that they take on an increasing amount of responsibility over the course of their clerkships. The literature on the subject of final-year clerkships mainly focuses on students’ learning, e.g. improving workplace based learning and assessment [see [2], [3], [4], [5]] or on the implementation of competency-based training [see [6], [7]]. Aspects of patient safety, on the other hand, have so far not been researched in depth. In this study, two central aspects of final-year clerkships are explored from the perspective of supervising physicians: current problems arising on the ward and possibilities for improvement through EPAs. 

In Germany, the Practical Year represents the final-year clerkship section which is divided into three specialist stages: internal medicine, surgery and an elective. The licensing laws for physicians (German: “ÄApprO”) stipulates that medical students at this stage of their training carry out professional activities assigned to them “according to their level of training, under the instruction, supervision and responsibility of a supervising physician” (§ 3, section 4, ÄApprO [8]). Additional detailed information is laid out in specific regulations and training content is detailed in so-called log books. There are no further stipulations as to how instruction and supervision are to be implemented in practice. This is also true for the aspect of patient safety. This therefore begs the question of how patient safety is ensured or indeed compromised during the final-year clerkships, and how risks for patients can be minimized. 

Patient safety is described by the* Aktionsbündnis Patientensicherheit* (German Coalition for Patient Safety) as the absence of adverse events [9]. Adverse events are defined as negative care results following medical care. An avoidable adverse event defines an event which is the result of an error [10]. To counteract problems caused by adverse events and avoidable adverse event, it is necessary to identify underlying errors. In doing so, possible strategies can be developed. Reason [11], in his error theory, describes two types of failure: failure caused by human error or violations (active failure), and errors caused through decisions at a management level, e.g. through cuts in resources (latent failure). Even when the effects of latent failure are not necessarily visible, they facilitate the incidence of active errors. Building on this theory, Woloshynowych et al. [12] developed an adapted framework for error analysis in the healthcare system by integrating error relevant factors from clinical practice.

A novel approach through which physicians’ training, and thus potentially patient safety, can be improved is in the implementation of training goals (outcomes) for the final year clerkship using Entrustable Professional Activities (EPAs). EPAs define specific medical professional activities entrusted to students or junior physicians gradually over the course of their training [13]. Activities with increasing scope and expertise are taken on by learners, with the degree of supervision decreasing while independency increases. EPAs are defined for different levels of training according to specific demands and limitations. There are five main levels of supervision: 1. allowed to observe, 2. direct supervision, 3. indirect supervision, 4. distanced supervision, 5. qualified to supervise others [14]. 

Additional, more detailed stages of supervision have been defined for undergraduate medical education [15]. The focus of EPAs so far has been on improving learning, and the potential significance of this concept for patient safety has not yet been investigated.

In this qualitative study, the subject of patient safety is explored from the perspective of supervising physicians. In the first section, problems perceived during the final-year clerkship are identified and in the second section, the views of supervisors on the potential for improvement through EPAs are analyzed. The study is based on the assumption that improving final-year clerkship training would mitigate latent sources of error and consequently lead to an increase in patient safety.

## 2. Methods

**Setting: **Data was collected using the qualitative method of focus group interviews at the Charité – Universitätsmedizin Berlin (Charité). The legal parameters for final year clerkships are provided by the ÄApprO [8] as well as local clerkship regulation. These stipulate that final-year clerks must “follow the instructions of training personnel” (§8, Practical Year regulation, Charité [16]). Training personnel have a specific duty of instruction and supervision. However, the type and scope of activities to be carried out are not further specified.

**Participants: **As part of a workshop on preparation for the “Habilitation” process (post-doctoral qualification procedure), 11 experienced physician specialists and consultants were recruited who supervise students in the surgery section of their final year clerkship (n=5) or in the internal medicine section (n=6). All of these prepared for the focus group by reading about the concept of EPAs and the specific activities defined for the undergraduate medical education. At the beginning of the workshop, there was further theoretical input on the concept of EPAs and a number of specific EPAs were discussed in more detail within the group. The subject of patient safety was not discussed beforehand. The focus groups were then separated into specialties and, led by A.C. and H.P., led through a structured interview (see attachment 1 ), recorded. The discussions within the focus groups lasted approx. 1.5 hours. Participation in the workshop was voluntary. Written consent was given for the analysis, approval by the data protection officer was deemed not necessary. 

**Analysis: **The audio recordings were transcribed and the data analysis was conducted in two stages using MAXQDA 18 software (VERBI Software, 2017, Berlin, Germany): firstly, in a primary analysis, relevant text passages were analyzed deductively with respect to the type and causes of problems arising during final-year clerkships. The categories are based on influencing factors in the clinical setting according to Woloshynowych et al. [12]: Task components, individual components, work conditions, organization and management, team components, patient components and finally the institutional context (see table 1 (Tab. 1)). The investigation into if and how EPAs can influence patient safety in the view of supervising physicians was carried out inductively [17]. In a methodical process, a system of categories was derived, with definitions and examples identified from the collected data. This was then used for the remaining data analysis. Both analyses were conducted by two independent raters (A.C. and Y.H.). When category allocations conflicted, a consenting process was used to agree on an allocation [17]. Two analysis cycles were carried out for each section. The results are presented as a description of the identified categories and the frequency with which they arose.

## 3. Results

In the following, the number of codings (mentions) of the respective categories as well as a summary description of the discussed contents are reported. Firstly, the perceived problems (contributory factors) are reported. This is followed by the estimated potential for improvement through EPAs. The error factors were discussed in both groups, but much more in the surgical focus group. The potential for improvement through EPAs was also discussed in both groups. However, the focus group for internal medicine focused more on these aspects. Within each group, the participants' contributions are distributed evenly among the topics discussed.

### Perceived problems in the final-year clerkship affecting patient safety

In the focus groups, the supervising physicians perceive various problems of the current training during final-year clerkships. Some of the described problems have a direct influence on patient safety, however most of them have an indirect effect. For example, poor clerkship conditions reduce the quality of education and patient care provided by students. All these factors can also be found and classified in the framework of factors influencing clinical practice described by Woloshynowych et al. [12]. 

Problems relating to the institutional context, the team or the patients are not mentioned in connection with the clerkship.

**Organization and Management **(34 mentions): The most intensively discussed problems relate to a lack of structure of the clerkship. The reason for this is mainly seen in the organization within the hospital operations. This is accompanied by the lack of consideration and recognition of the supervision and training of students. It is worth noting that the structural problems are usually described in combination with and as the cause of other problems. Due to the frequency and detail of their mentions, as well as their influence on other categories, problems of this factor appear most serious (see table 2 (Tab. 2)).

**Individual (staff) factors **(24 mentions): This includes mainly motivational problems: From the supervisors’ perspective this is often related to structural problems. For the students, they mention excessive demands as the cause of insufficient motivation. They also discuss inaccurate self-assessment of one's own abilities as a cause for avoidable adverse events.

**Task factors** (8 mentions): Problems can arise if assigned tasks are inappropriate or cannot be fully verified. An example of this is communication with patients, which is perceived as problematic. Careless comments by students may have a significant influence, such as the willingness to undergo further care on the part of the patient. In this category, the reciprocal instruction and supervision of students, advocated by the physicians, is also discussed: peer supervision. For certain medical procedures (e. g. introducing a venous catheter), a work culture has become established in which the students teach such procedures themselves or supervise each other – supervision by a physician is omitted.

**Work environment components** (7 mentions): Other problems mentioned are assigned to the factor “work environment”. These relate primarily to a high workload and the resulting lack of time.

#### Improvement on patient safety by EPAs during final-year clerkship

The discussion on the influence of EPAs on patient safety shows a consistently positive response among supervising physicians. The aspects found in their statements can be summarized into a category system with four overall, distinct categories (see table 3 (Tab. 3)). The supervising physicians agree that EPAs can improve the final-year clerkship and, as a result, increase patient safety.

**Improvement of training** (49 mentions): In the view of supervising physicians, the use of EPAs would in particular lead to a more structured clerkship and therefore a fundamental improvement in medical training. This would lead to an increase in patient safety. Though an obligatory training catalogue - similar to a curriculum for the final year clerkship - content would be less random and thus less dependent on the motivation of the final-year clerkship students and their supervisors. This alone would lead to improved training at the end of the undergraduate medical training and subsequently to better qualified physicians starting out in their career. If newly qualified physicians worked at the level of competency of a ward physician, this would lead to an immediate improvement in patient safety. The standardization of learning content would be accompanied by a prioritization and better organization of training on the ward. If the supervision of students happens not only on the fly but with clear goals and appropriate resources, this would contribute to a general improvement of training. Patients would also benefit by being treated by better trained physicians.

**Performance levels and support of learning processes** (16 mentions): Supervising physicians see advantages of EPAs not only for course learning objectives (outcomes), but also for the entire final year clerkship. EPAs can help provide an overview of each student’s performance level over the course of the clerkships. The requirements of the clerkship and the level to which these should be fulfilled become clearer for both students and supervisors.

**Transparency** (13 mentions): The entire final-year clerkship would become more transparent, as expectations of students are written down and binding. In addition, clinics and hospitals who train final-year clerkship students can be evaluated based on the outcomes achieved. It would be clearer to patients that they were being treated in a university clinic or a teaching hospital. Through obligatory EPAs, they would also be able to judge which activities final year clerks are qualified to carry out. Transparent requirements with regard to the expected learning level would have a positive impact on students’ learning.

**Minimizing uncertainty** (8 mentions): Important effects on legal-formal issues and security in action were identified. A concrete description of the required (and thus permitted) task together with the necessary level of supervision would give supervising physicians the confidence to know which activities they can delegate to final-year clerkship students. If they overstep their abilities, a binding framework for supervising physicians and for patients offers legal assurance. Final-year clerkship students, meanwhile, are clearly informed about which activities they are permitted to carry out, which will help to minimize uncertainty when they are dealing with patients.

## 4. Discussion

The analysis of current sources of error in final year clerkships reveals that in particular, the lack of operationalization in training leads to problems with patient safety. The absence of a standardized, binding structure means that at the end of their training, medical students cannot all be expected to have the same competencies in carrying out certain tasks independently. Without binding guidelines, the content and scope of training during final-year clerkships comes down to the motivation of physicians and students. This leads to heterogeneous training quality, largely dependent on individual factors which can also influence the quality of patient care. This results in a variety of problems for patient safety. Poor communication can disconcert patients and negatively influence their decisions to undergo further treatment. Potential errors in the instruction and execution of medical procedures under peer-supervision seem obvious. This aspect is particularly remarkable as this way of doing things is seen as normal and unproblematic by supervising physicians (see table 1 (Tab. 1)). However, this can have a significant influence on patient safety. “Catheter and tubing mis-connections” are an example of general problems for patient safety listed by the WHO [18] as having a high potential for danger. The fact that students are teaching themselves such skills seems questionable. The legal aspects of avoidable adverse events are also unclear in this instance. Uncertainty on the part of supervising physicians also seems to be a fundamental aspect: in daily life on the ward, it is often unclear whether and to what degree students have already received training in concrete activities by other colleagues. Professional activities may be assigned to students without any kind of check on whether they can actually master them.

In summary, an important lesson of this investigation is that problems arising during clerkship training are generally caused by a lack of training structure, both in content and organization. Failures caused by human error are rare occurrences which are not perceived as a fundamental problem of final-year clerkships. Rather, the problems described relate to latent sources of error [12] which could be reduced by the introduction of EPAs.

Despite the potential for maximizing patient safety for final-year clerkships, there is still a lack of clear and standardized requirements at many German universities. We can assume that a more clearly structured training phase would not only benefit students and supervising physicians, but would also have a positive effect on patient safety. With better trained graduating physicians who are able to work more independently on the ward, it can be assumed that on the whole there will be fewer errors. Even during clerkship training, patient safety can already be improved. If there is transparency on which activities students are able and permitted to perform under what degree of supervision, there are clear boundaries. This leads to improved safety in routine processes.

With regards to method, it should be noted that both systems of category used for the data analysis were appropriate. The inductive category system for the analysis of potential improvement of patient safety through EPAs proved to be a suitable method. All problems described by supervising physicians were correlated to four of the seven factors described by Woloshynowych et al. [12]. As expected, problems connected to patient personality or the institution itself are not associated with the training level of final-year clerkship students so that there were no mentions in these categories. For problems of team work, the level of training may be a possible cause. The lack of mentions may be due to the small sample number (n=11), so that we cannot assume a saturation of all categories.

A further limitation of this study is that, due to its exploratory nature and the selective sample, the results are not necessarily generalizable to final-year clerkships beyond the Charité. Even here, they may not be transferable to other specializations without further review. In particular, during the elective stage of clerkships, the higher motivation of students and supervising physicians may lead to differing perceptions and assessments. It would also be interesting to investigate the perspective of students and how they perceive their clerkships with respect to patient safety and whether this could be improved by the implementation of EPAs

## 5. Conclusions

In the literature, EPAs have been described primarily from the curricular perspective as a way to improve learning. This study opens up a wider perspective in which EPAs may be implemented as part of workplace-based learning. EPAs may improve patient safety by providing structure to final-year clerkships with more clearly defined binding requirements and thereby creating transparency for all being involved.

## Competing interests

 The authors declare that they have no competing interests. 

## Supplementary Material

Structured interview for focus groups on the topic: Assigning activities on the ward to students on final year clerkship

## Figures and Tables

**Table 1:**
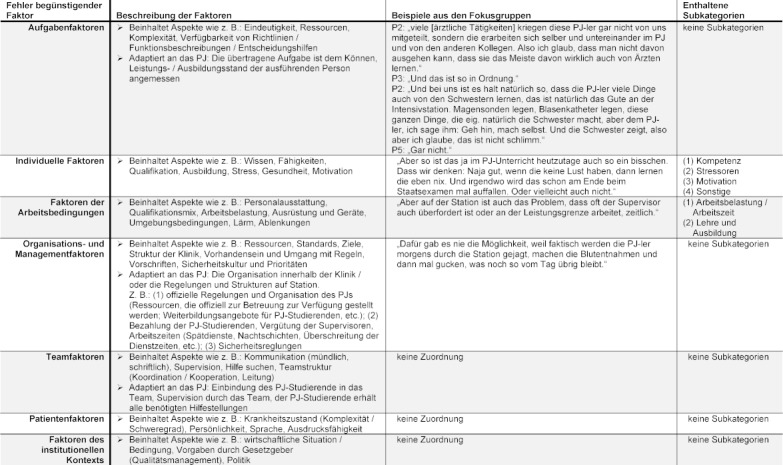
Contributory factor (adapted from Woloshynowych et al. [12]). Representation of the categories with anchor examples from the focus groups.

**Table 2:**
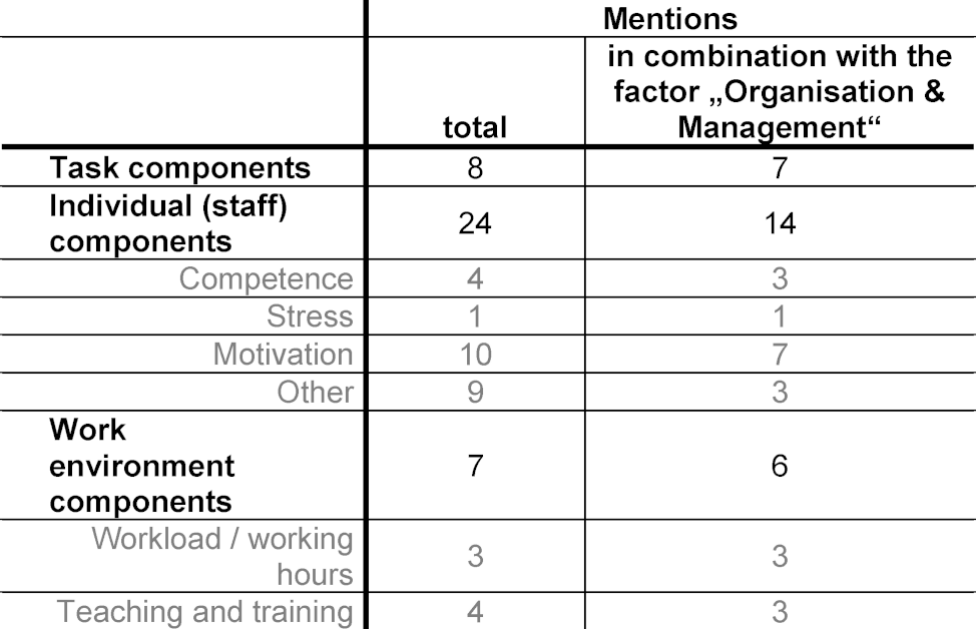
Connections between Contributory factors. Connections between contributory factors. Number of simultaneous mentions of the category Organization & Management with other problems.

**Table 3:**
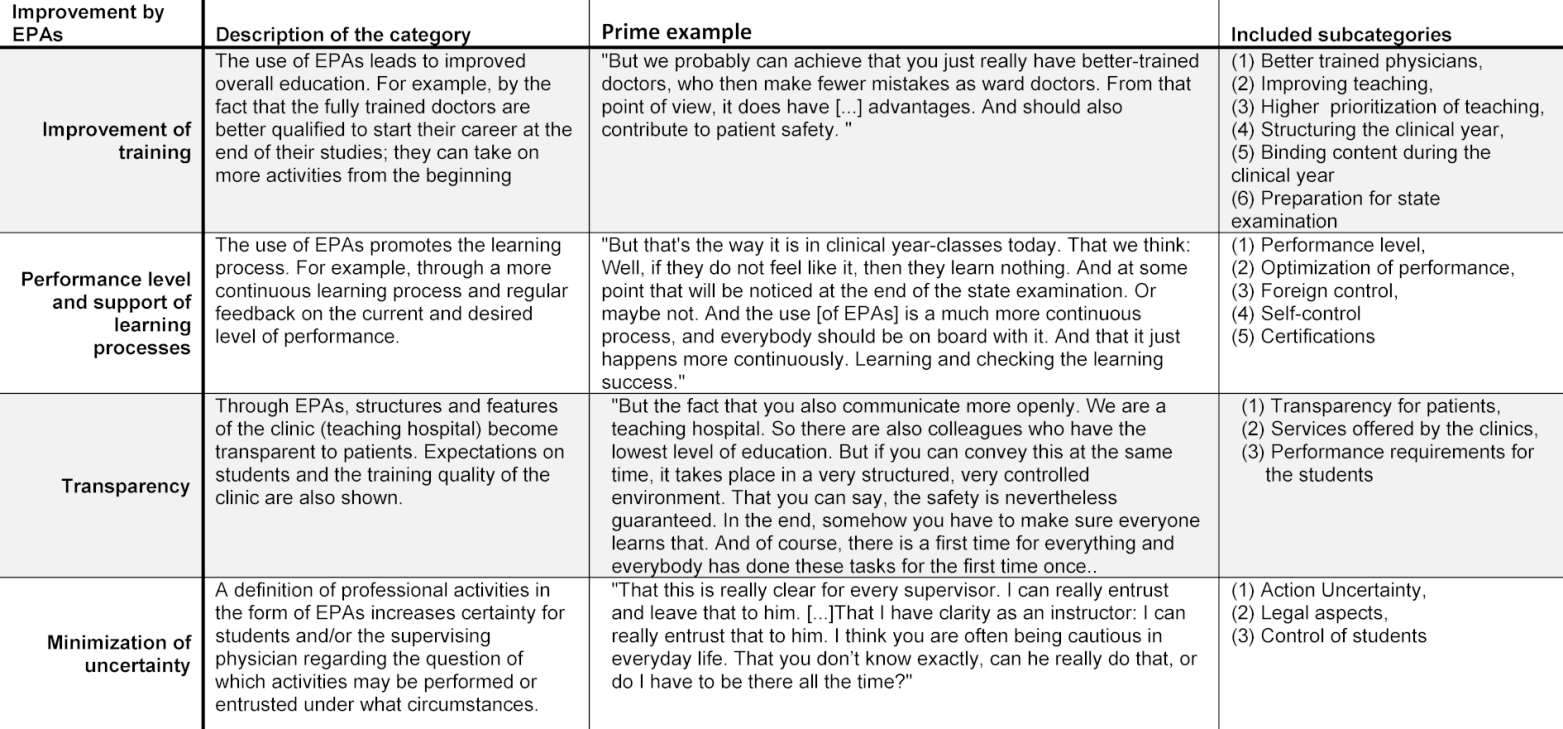
Category system of the “Improvement by Entrustable Professional Activities (EPAs)”. Overview and description of the main categories.
