# Supplementary material for: Patient safety during final-year clerkships: A qualitative study of possible error sources and of the potential of Entrustable Professional Activities
Source: GMS J Med Educ. 2019 Mar 15;36(2):Doc18. doi: 10.3205/zma001226 (PMC6446462; doi:10.3205/zma001226)
Supplement: Structured interview for focus groups on the topic: Assigning activities on the ward to students on final year clerkship [file JME-36-2-18-s-001.pdf]

Structured interview for focus groups on the topic: Assigning activities on the ward to students on final year clerkship

(1) How do you regulate the assuming of activities by students in their clinical year in the ward operation?

(2) What difficulties or problems arise when students take on professional activities on the ward?
   a. Please describe what causes the difficulties and / or problems.
   b. Please evaluate if these difficulties have implications for patient safety.

(3) Please assess if EPAs are appropriate as a way of selecting appropriate professional activities for students on the ward.
   a. Discuss the advantages and disadvantages of the EPA concept for assigning activities on the ward to final-year clerkship students.
   b. Evaluate the advantages and disadvantages compared to your previous approach to selecting activities for students on the ward.
